# Supplementary material for: Black carbon and dust alter the response of mountain snow cover under climate change
Source: Nat Commun. 2022 Sep 20;13:5279. doi: 10.1038/s41467-022-32501-y (PMC9489766; doi:10.1038/s41467-022-32501-y)
Supplement: Supplementary file 1 — Supplementary Information [file 41467_2022_32501_MOESM1_ESM.pdf]

Black carbon and dust alter the response of mountain snow cover under climate change

Marion Réveillet [1*], Marie Dumont [1*], Simon Gascoin [2], Matthieu Lafaysse [1], Pierre Nabat [3], Aurélien Ribes [3], Rafife Nheili [1], Francois Tuzet [1], Martin Ménégoz [4], Samuel Morin [3], Ghislain Picard [4], Paul Ginoux[5]

[1]Univ. Grenoble Alpes, Université de Toulouse, Météo-France, CNRS, CNRM, Centre d'Etudes de la Neige, 38000 Grenoble, France
[2]Centre d'Etudes Spatiales de la Biosphère (CESBIO), Université de Toulouse, CNRS/CNES/IRD/INRAE/UPS, 31400 Toulouse, France
[3]CNRM, Université de Toulouse, Météo-France, CNRS, Toulouse, France
[4]Univ. Grenoble Alpes, CNRS, IRD, IGE, 38000 Grenoble, France
[5]NOAA Geophysical Fluid Dynamics Laboratory, 201 Forrestal Road, Princeton, NJ 08540, USA

*To whom correspondence should be addressed; E-mail: marie.dumont@meteo.fr or

marion.reveillet@univ-grenoble-alpes.fr

Supplementary material

Supplementary text

A. ANOVA equations

Following Eq. 6 in Geoffroy et al., *(32)*, the SMOD, noted S, can be decomposed as a sum of one-variable functions and an interaction term I, referred to as a ”sequencing” term:

$$S = p_0 + p_1(\text{meteo}) + p_2(\text{LAP}) + I(\text{meteo}, \text{LAP}) \tag{1}$$

where p_0 is constant, p_1 depends on the meteorological conditions only, p_2 depends on LAPs deposition fluxes only and I includes interactions between meteorological conditions and LAPs deposition, i.e., the fact that the response to given LAP deposition can depend on meteorological conditions. For example, dust might be directly buried after deposition if a sufficiently large snowfall event occurs immediately after dust deposition.

The contribution, $\hat{c}$, of each function p_1, p_2 to the variance of the SMOD is computed following the equations (2) and (3), where N_1 denotes the number of values taken by the meteorological conditions (39 individual years) and $N_2 = 2$ (simulations with or without LAPs). i,j are the parameter values. The interaction term ($\hat{c_I}$) (or sequencing) is computed following the equation (4).

$$\hat{c}(p_1) = \frac{1}{var(S)} \frac{1}{N_1} \sum_{i=1,N_1} \left[\frac{1}{N_2} \sum_{j=1,N_2} (S_{i,j} - \overline{S}) \right]^2 \tag{2}$$

$$\hat{c}(p_2) = \frac{1}{var(S)} \frac{1}{N_2} \sum_{i=1,N_2} \left[\frac{1}{N_1} \sum_{j=1,N_1} (S_{i,j} - \overline{S}) \right]^2 \tag{3}$$

$$\hat{c}(I) = 1 - (\hat{c}(p_1) + \hat{c}(p_2)) \tag{4}$$

B. Past trends

The simulated SMOD trend is generally significant with a mean decrease of 7.7 days per decade (corresponding to a decrease of 3.9% of the snow season duration) considering all the North Alps and 5.7 days per decade (3.0%) for the South Alps. As for results presented at 2100 m a.s.l. this decrease is consistent with positive trends in air temperature (i.e. significant temperature trends ranges from 0.1 to 0.3°C per decade (Supplementary Fig. S7). The SMOD trend simulated in the Pyrenees is lower (i.e., a decrease of

5.5 and 6.0 days per decades for the North and South Pyrenees respectively, corresponding to a decrease of 3.2 and 3.1% of the snow season duration) and also consistent with trends in air temperature.

When accounting for BC and dust, the average SMOD trend is 5.4 days per decade for the North Alps (corresponding to a decrease of 3% of the snow season duration) 4.1 for the South Alps (2.4%), 3.4 for the North Pyrenees (2.2%) and 5.5 for the South Pyrenees (3.1%) (Supplementary Fig. S7). The effect of the negative trend of BC deposition observed at 2100m a.s.l. is here confirmed for all the Alps and Pyrenees.

C. Model evaluation

Observed vs simulated snow cover area

The evaluation is done in comparing daily simulated snow cover area (SCA) to MODIS SCA for $S_{BC+Dust}$, S_{pure}, and $S_{baseline}$, over the winter period (from November to June). Four metrics methods are used: R^2, RMSE, bias, and Jaccard index (*J*). *J* is defined by the number of pixels that are snow covered in both simulation (*S*) and observation (*O*) areas, divided by the total number of pixels in the union of *S* and *O*; and ranges between 0 and 1, 1 meaning *S*=*O*.

Supplementary Fig. S11 shows the scores for each massif, when comparing observed SCA (from MODIS) and simulated SCA ($S_{BC+Dust}$, $S_{baseline}$ and S_{pure}), computed over the period 2000–2016, considering only the winter period (i.e., November to June). For bias and J, the mean is reported. Despite low and not significant differences for R^2 and J between the four simulations, for both the Alps and the Pyrenees, the explicit representation of BC and dust ($S_{BC+Dust}$) leads to a significant decrease in bias as well as a lower RMSE (Supplementary Fig. S11).

Comparison to local measurements

An evaluation is done in terms of daily snow depth variation. The daily snow depth variation ΔSD_n is defined for n days as:

$$\Delta SD_n = SD_n - SD_{n-1} \tag{5}$$

Then, the evaluation is done on the snow melt. For that purpose only negative ΔSD_n are kept and compared to the daily simulated snow melt in order to conserve only values when snow melt occurs (ΔM_n). Finally, daily bias are computed between the observed and simulated ΔM_n for each simulations

(i.e. ($S_{BC+Dust}$, $S_{baseline}$ and S_{pure}) (Supplementary Fig. S12).

Supplementary figures

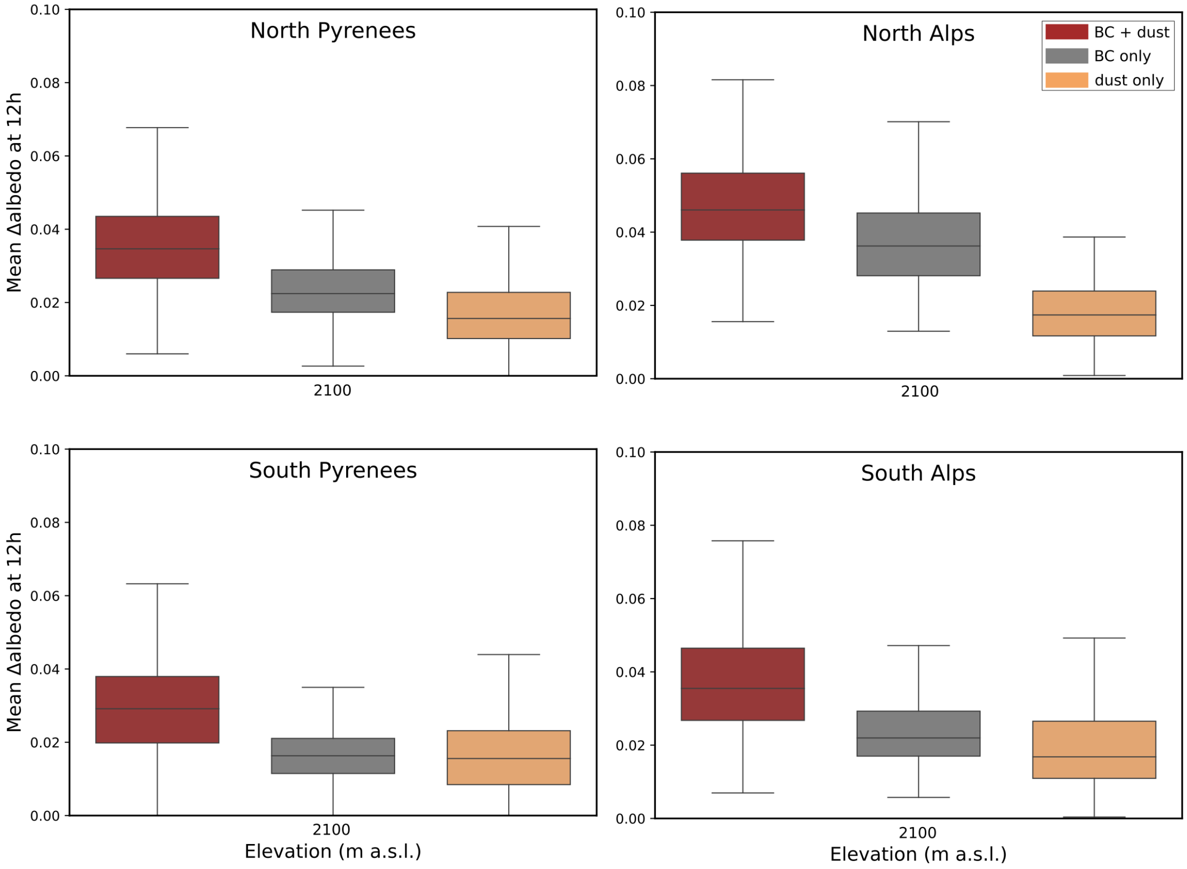

Figure S1: **Broadband albedo changes**. Differences between pure and impure simulations (i.e. BC + dust, only BC and only dust) of the mean albedo at 12h - local time. The differences are computed at 2100 m a.s.l., considering the entire study period (i.e., 1978-2018). Daily differences are computed over the snow season (i.e for days with snow on the ground) and the average of the annual difference is used for the figure. The boxes show the quartiles of the distribution corresponding to the inter-annual and spatial variability. Minimum/maximum ranges (excluding outliers) are indicated by the whiskers.

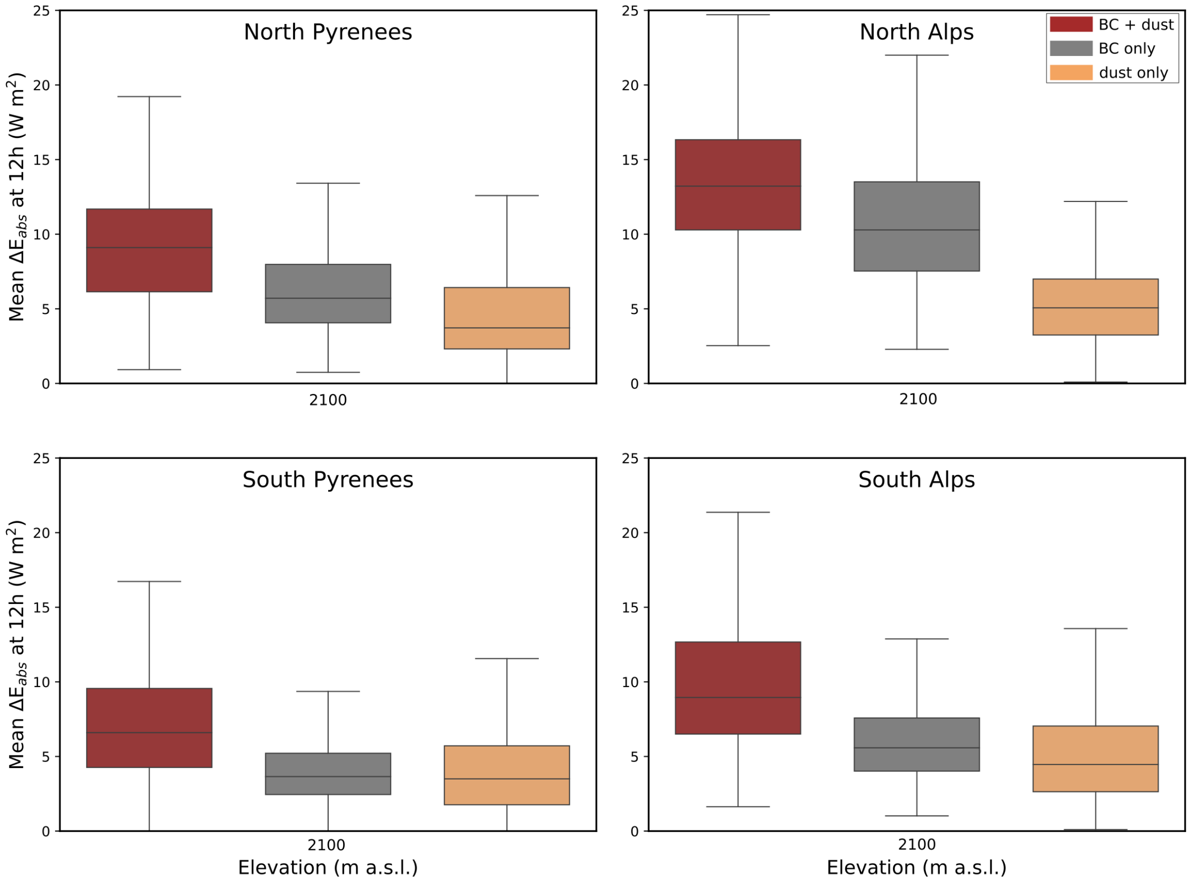

Figure S2: **Radiative impacts**. Differences between pure and impure simulations (i.e. BC + dust, only BC and only dust) of the mean solar energy absorbed at 12h. The differences are computed at 2100 m a.s.l., considering the entire study period (i.e., 1978-2018). Daily differences are computed over the snow season (i.e for days with snow on the ground) and the average of the annual difference is used for the figure. The boxes show the quartiles of the distribution corresponding to the inter-annual and spatial variability. Minimum/maximum ranges (excluding outliers) are indicated by the whiskers.

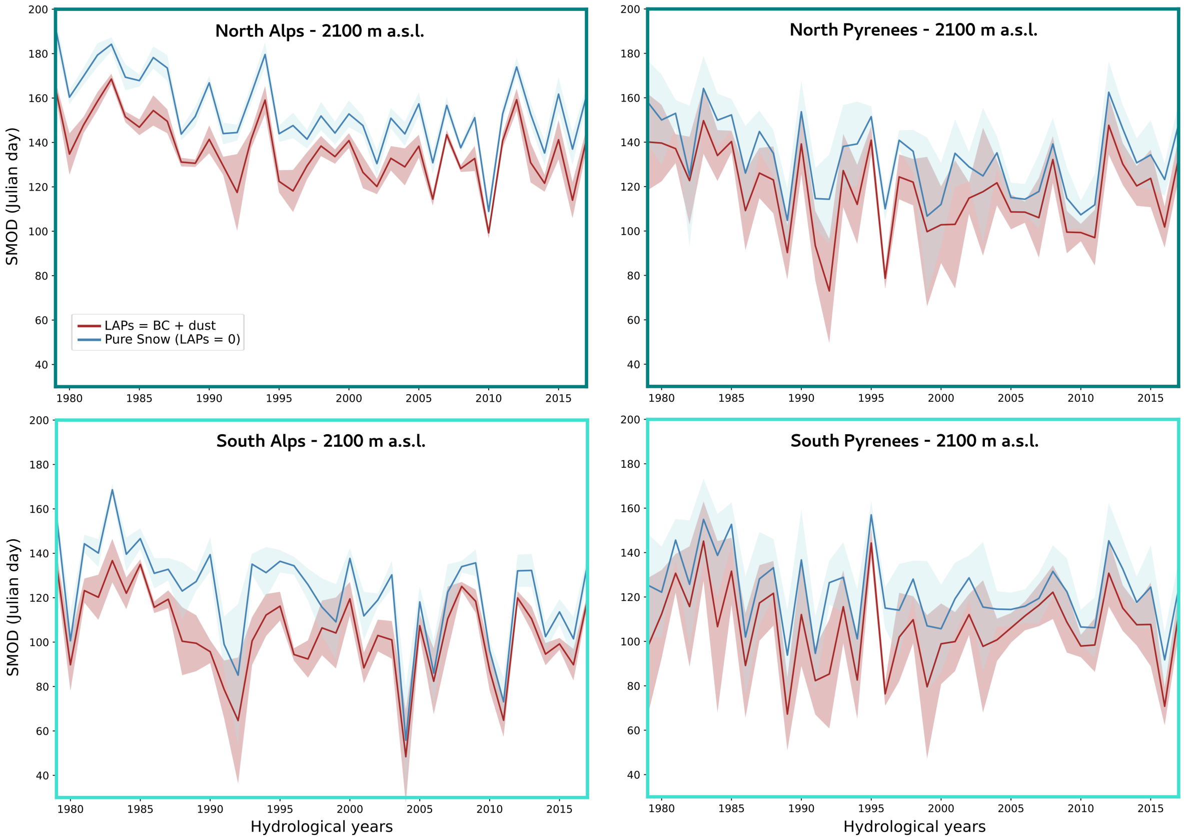

Figure S3: **Simulated snow melt-out date.** Temporal evolution of the simulated snow melt out date (solid lines) over the 1979–2018 period, at 2100 m a.s.l., with the standard deviation (shaded areas, representing the spatial variability). Pure simulations are represented in blue and simulations considering the effect of BC and dust are in red.

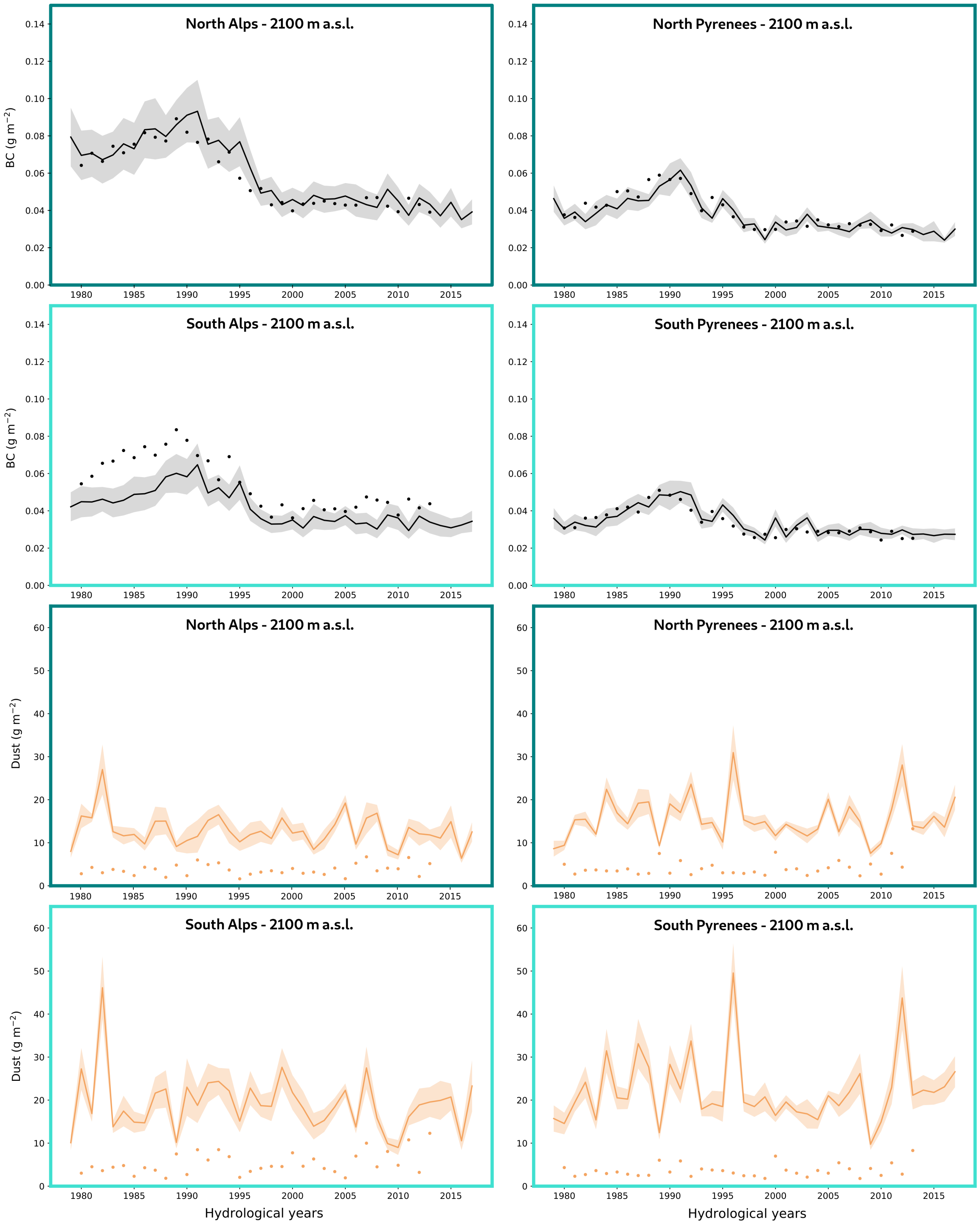

Figure S4: **Annual BC and dust deposition.** Temporal evolution of the mean annual cumulative BC (grey) and dust (orange) deposition from CNRM-ALADIN62 model (solid lines) and for the 1979-2018 period, at 2100 m a.s.l., with the standard deviation (shaded areas, representing the spatial variability). Temporal evolution of the mean annual cumulative BC and dust deposition from GFDL-AM4 model for the 1980-2014 period is reported by the points.

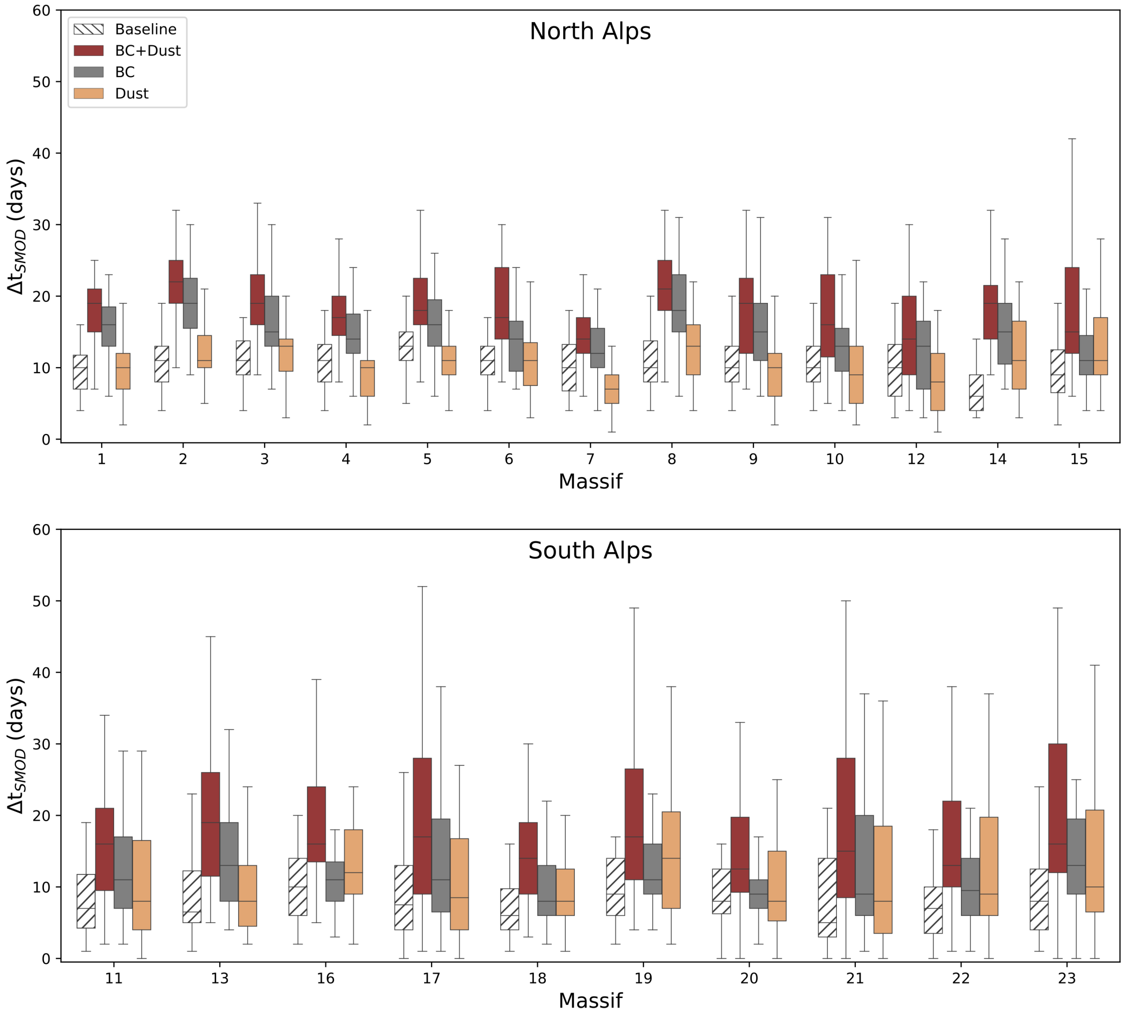

Figure S5: Shortening of the season computed as the difference in snow melt-out date (SMOD) between the pure snow simulations and simulations considering BC and dust (red), dust only (orange), BC only (grey) and considering implicitly the LAPs (i.e. an albedo decrease based on the snow age only, baseline, white hatched). SMOD differences are computed at 2100 m a.s.l., considering the entire study period (i.e., 1978-2018), for each individual SAFRAN massif in the French Alps- see Fig. S11. The boxes show the quartiles of the distribution corresponding to the inter-annual variability. Minimum/maximum ranges (excluding outliers) are indicated by the whiskers.

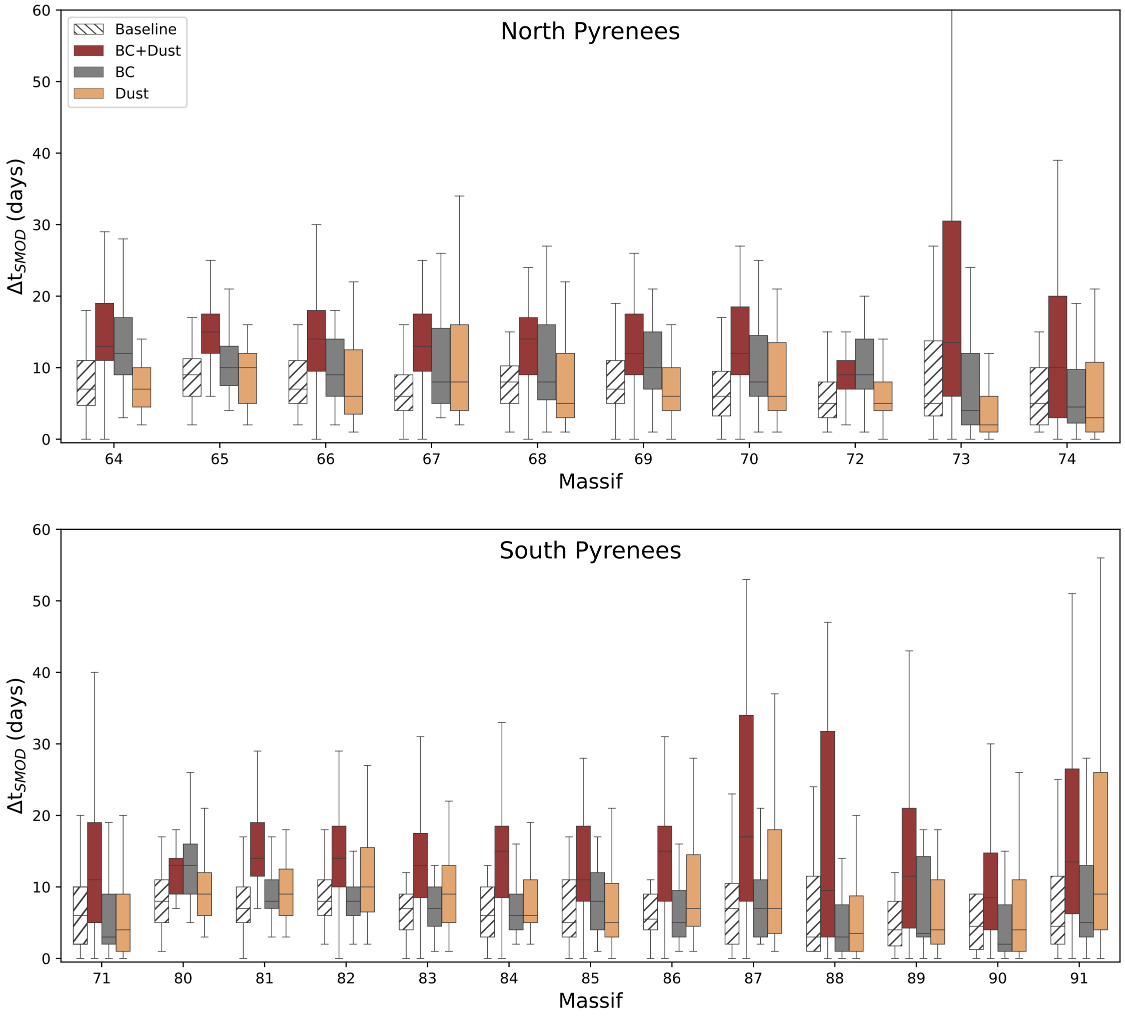

Figure S6: Shortening of the season computed as the difference in snow melt-out date (SMOD) between the pure snow simulations and simulations considering BC and dust (red), dust only (orange), BC only (grey) and considering implicitly the LAPs (i.e. an albedo decrease based on the snow age only, baseline, white hatched). SMOD differences are computed at 2100 m a.s.l., considering the entire study period (i.e., 1978-2018), for each individual SAFRAN massif in the Pyrenees - see Fig. S11. The boxes show the quartiles of the distribution corresponding to the inter-annual variability. Minimum/maximum ranges (excluding outliers) are indicated by the whiskers.

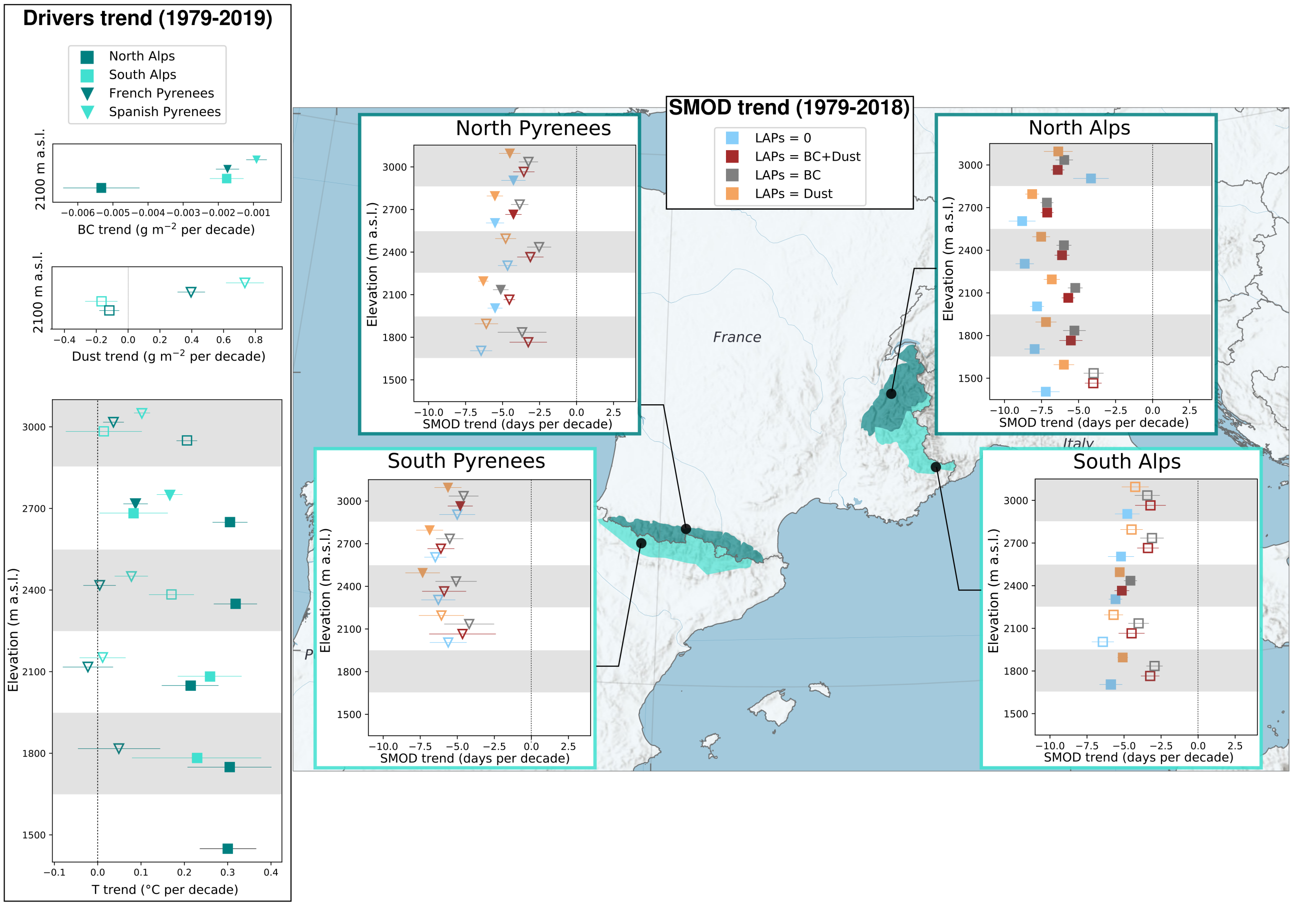

Figure S7: **Past trends.** Trends in air temperature (at 2m above the surface), BC and dust deposition and SMOD of temporal series computed from annual values over the period 1979–2018, as a function of elevation. Trends are represented as the the best estimate and 90% confidence range, per area (North Alps, South Alps, North Pyrenees and South Pyrenees). Only markers of significant trends (t-test 0.05) are filled in. Errors bars represent the spatial variability. Only elevations with a mean SD >30 cm over the winter period (i.e. 1st of December to 30 of April) are represented.

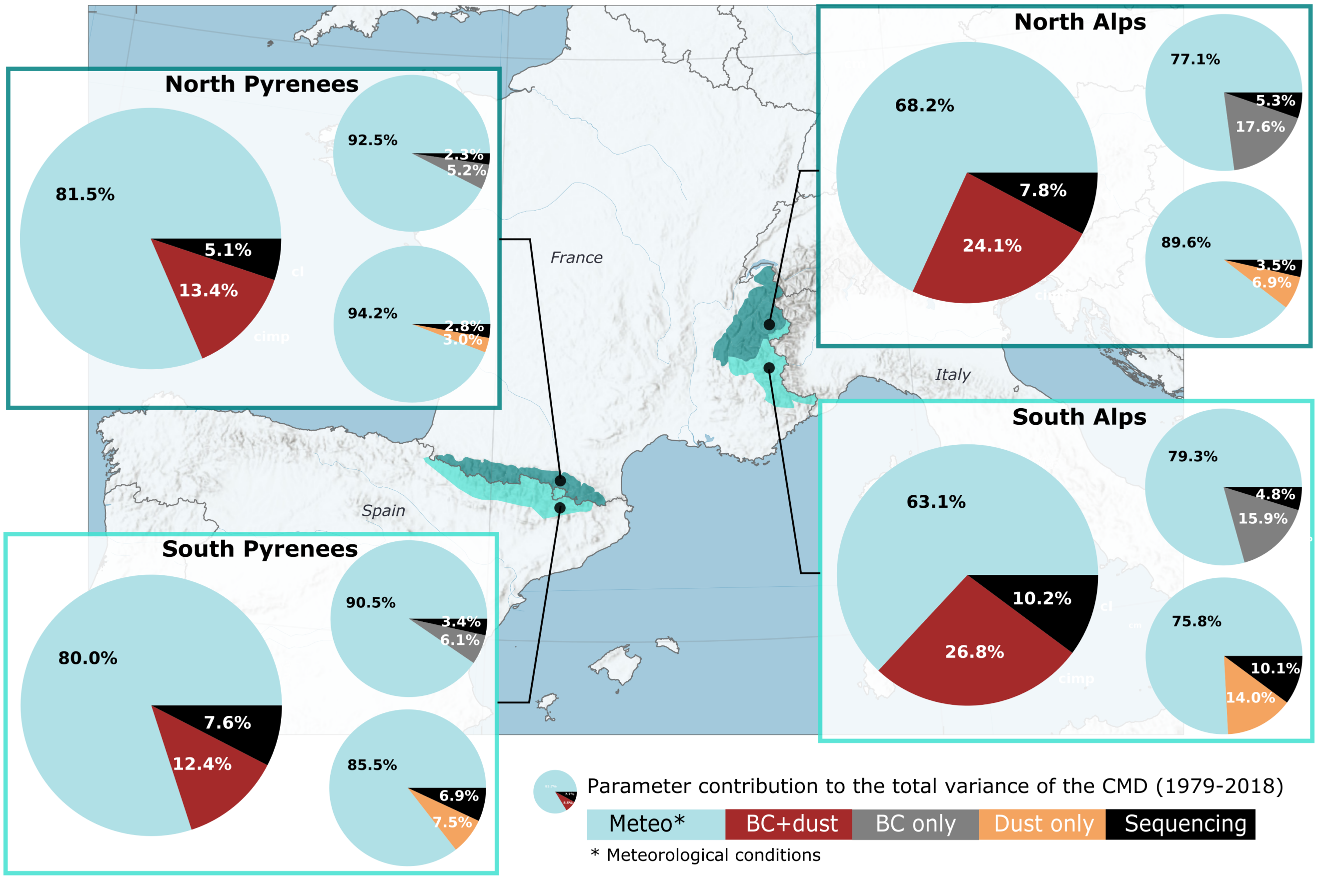

Figure S8: **BC and dust contribution to the snowmelt runoff.** Contribution of the meteorological conditions and BC and dust to the variance of the CMD (in days) for the North and South Alps (left), North and South Pyrenees (right) computed over the 1979–2018 period at 2100 m a.s.l.. Larger circle indicate contributions of the parameters considering the role of BC+Dust (brown) and smaller circle represent the contribution of BC (grey) and dust (orange) separately. The intersection term (purple) indicate the contribution of BC and dust and meteorological forcing, and is due to the dependence of the BC and dust contribution to the meteorological conditions.

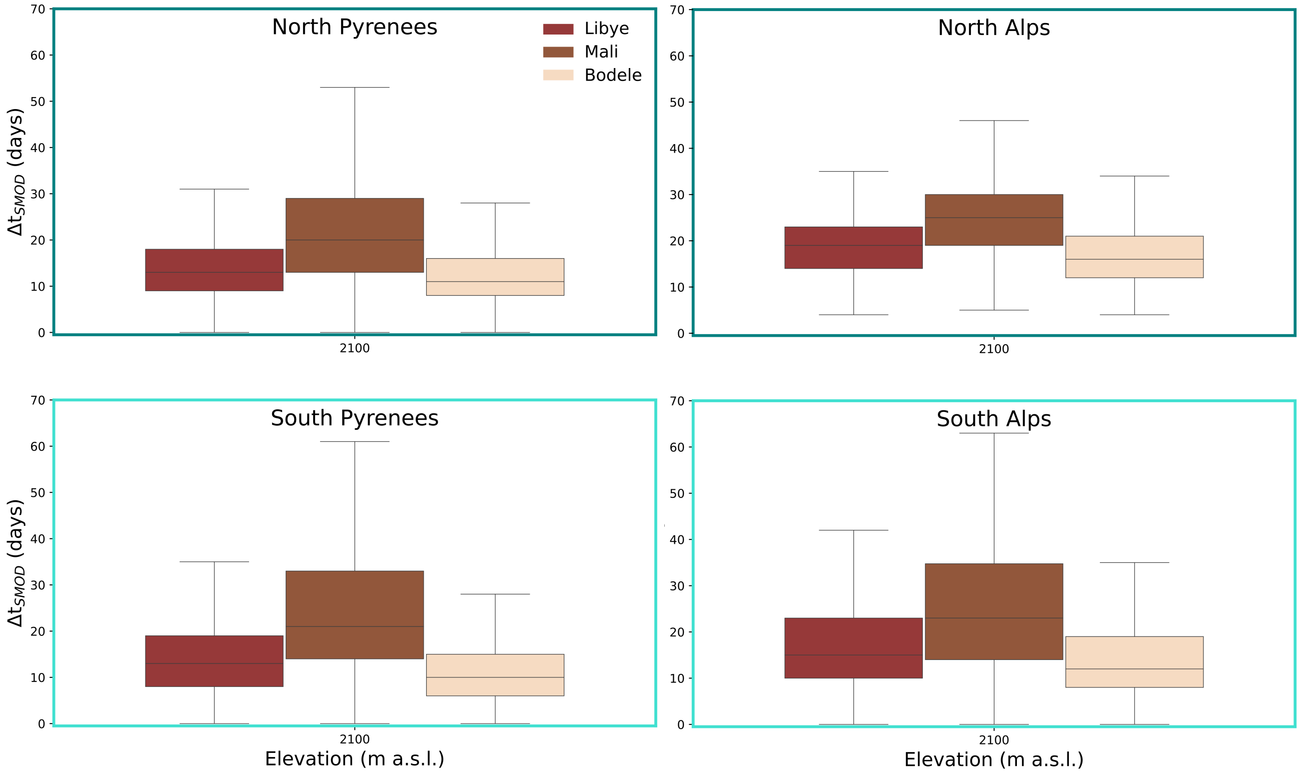

Figure S9: **Sensitivity to dust absorption coefficient.** Shortening of the season computed as the difference in snow melt-out date (SMOD) between the pure snow simulations and simulations considering BC and dust for different dust spectral signatures (i.e. a mass absorption efficiency at 400nm of 27 10^{-3} m^2 g^{-1} (Bodele), 110 10^{-3} m^2 g^{-1} (Libye, used in this study) and 630 10^{-3} m^2 g^{-1}(Mali)). SMOD differences are computed at 2100 m a.s.l., considering the entire study period (i.e., 1978–2018), for the North and South Pyrenees (left) and the North and South Alps (right). The boxes show the quartiles of the distribution corresponding to the inter-annual and spatial variability. Minimum/maximum ranges (excluding outliers) are indicated by the whiskers.

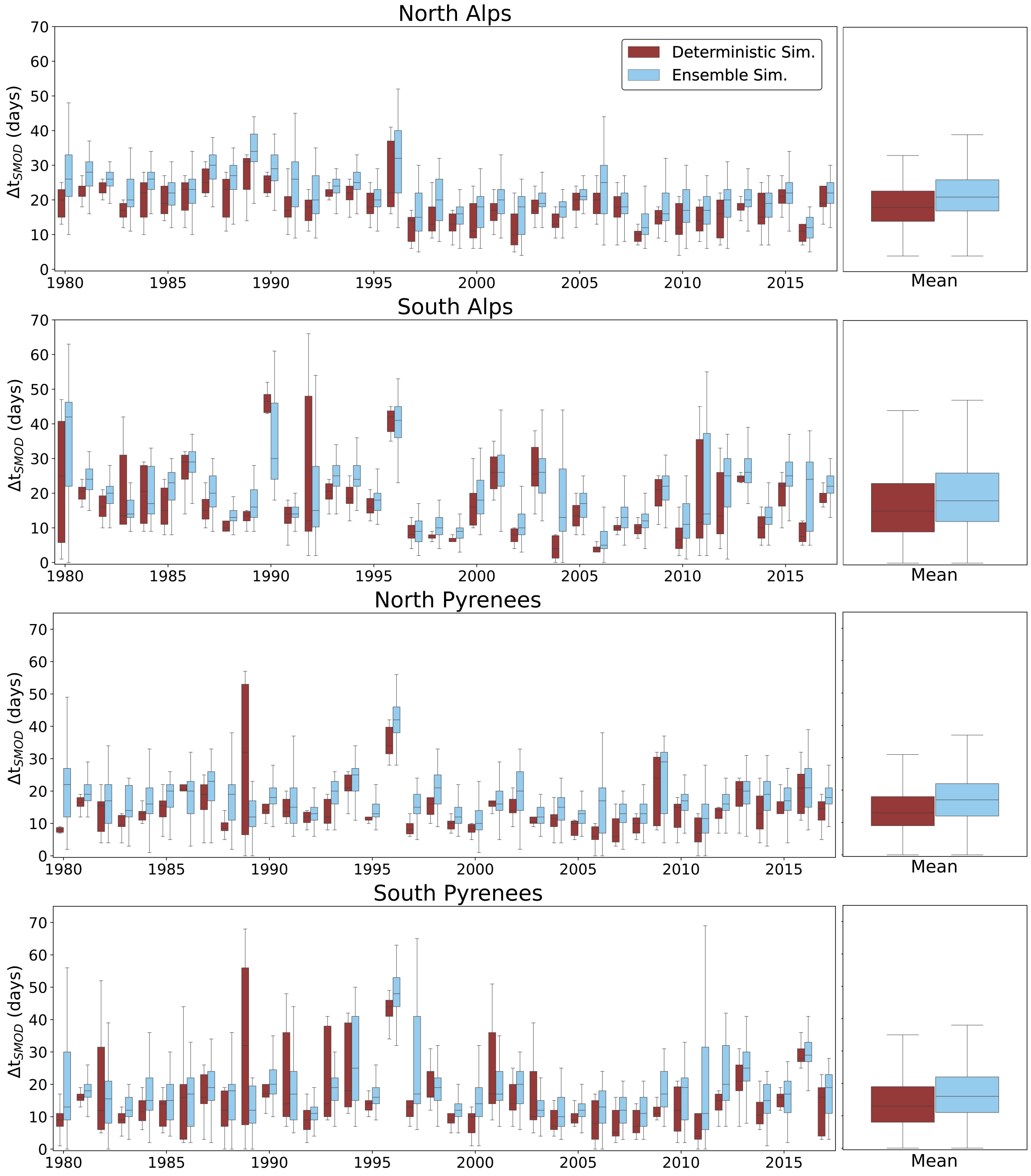

Figure S10: **Uncertainty in snow physic representation.** Shortening of the season at 2100 m a.s.l. computed as the difference in snow melt-out date (SMOD) between the pure snow simulations considering BC and dust, as a function of the year (left), and averaged for all elevation (right), for the four studied areas (North and South Alps, North and South Pyrenees), over the 1979–2018 period. For the deterministic simulations (brown), the boxes show the quartiles of the distribution corresponding to the spatial variability. For the ensemble simulations (blue), the boxes show the quartiles of the distribution corresponding to both the spatial variability and the uncertainty in snow physic representation. Minimum/maximum ranges (excluding outliers) are indicated by the whiskers.

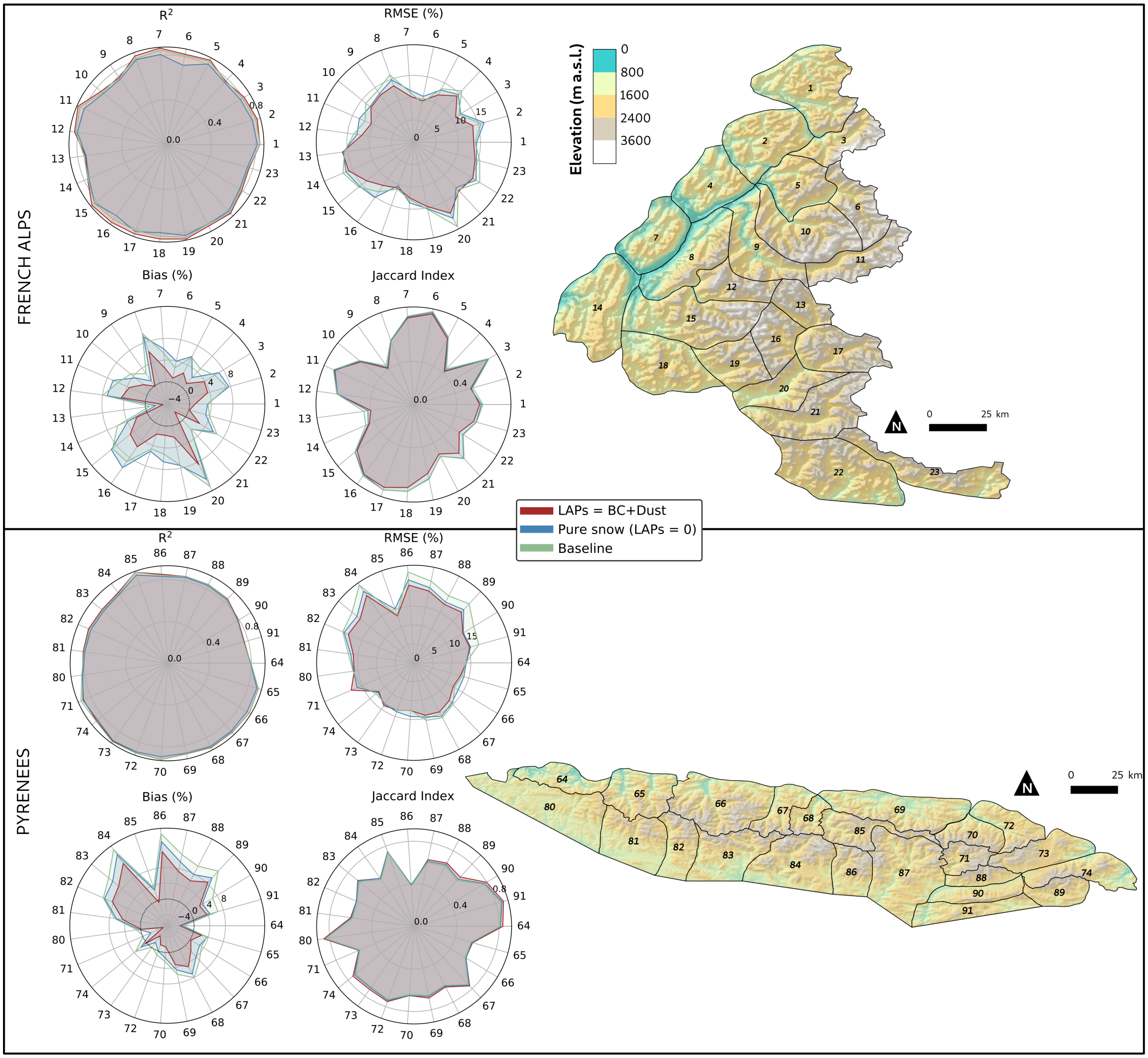

Figure S11: **Model evaluation using satellite images.** Scores computed over the 2000–2016 period, between the daily observed (MODIS) and simulated SCA for $S_{BC+Dust}$ (brown), S_{pure} (blue) and $S_{baseline}$ (green). Each massif of the Alps are represented by the upper panels and the Pyrenees by the lower ones. The massif number locations are reported in the maps on the right

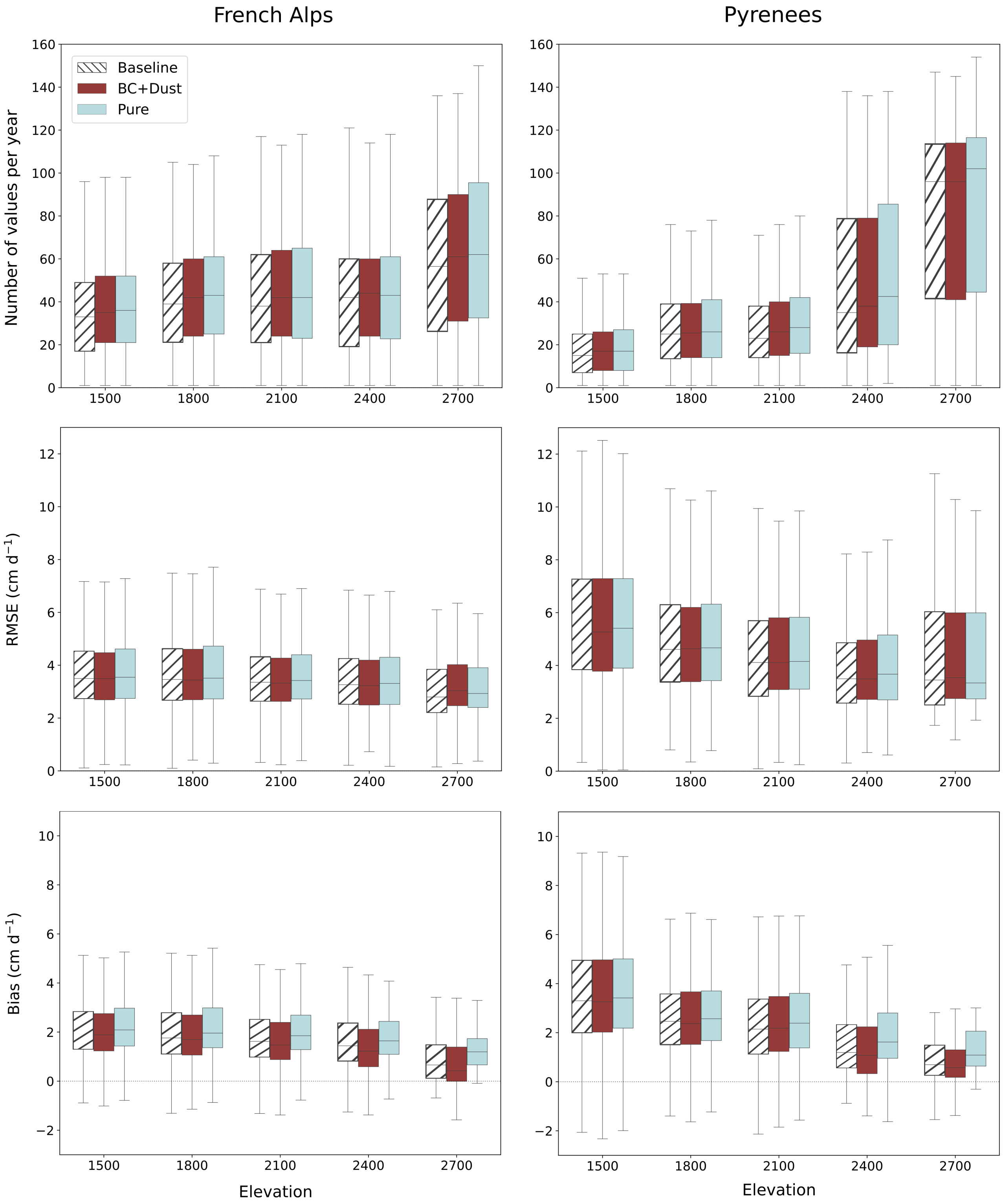

Figure S12: **Model evaluation using station measurements.** Number of values, RMSE and bias computed over the 1983–2018 period, between the daily observed corresponding to melt and simulated ΔSD corresponding to melt for $S_{BC+Dust}$ (brown), S_{pure} (blue) and $S_{baseline}$ (white, hatched). Results are presented by elevation range for the French Alps (left) and the Pyrenees (right) separately.

Supplementary Tables

Simulations	Scores	All elevations
Baseline	RMSE (days)	19.4
	MAE (days)	**12.8**
	IQ range (days)	**16.0**
	Bias (days)	3.6
	# Values	788
BC + dust	RMSE (days)	**19.2**
	MAE (days)	13.3
	IQ range (days)	18.5
	Bias (days)	**-0.68**
	# Values	803
Pure	RMSE (days)	23.6
	MAE (days)	17.0
	IQ range (days)	21.5
	Bias (days)	12.4
	# Values	715

Table S1: **SMOD evaluation at the snow-depth measurements stations.** Scores between simulated SMOD and observed SMOD from 495 stations located in the French Alps and Pyrenees. Numbers in bold indicate the best score. blackFour metrics are indicated, the root mean square error (RMSE), the mean absolute error (MAE), the interquartile range (IQ) and the bias (model-observation). The interquartile range is given as the distance between 25% and 75% quantiles of the error.
